# A Data Set of Signals from an Antenna for Detection of Partial Discharges in Overhead Insulated Power Line

**DOI:** 10.1038/s41597-023-02451-1

**Published:** 2023-08-21

**Authors:** Lukáš Klein, Jan Fulneček, David Seidl, Lukáš Prokop, Stanislav Mišák, Jiří Dvorský, Marian Piecha

**Affiliations:** 1 Department of Computer Science, VSB - Technical University of Ostrava, Ostrava, Czech Republic; 2 ENET centre - CEET, VSB - Technical University of Ostrava, Ostrava, Czech Republic; 3 MINISTRY OF INDUSTRY AND TRADE, Prague, Czech Republic

**Keywords:** Power distribution, Scientific data, Electrical and electronic engineering

## Abstract

We introduce a data set obtained via a contactless antenna method for detecting partial discharges in XLPE-covered conductors used in medium-voltage overhead power transmission lines. The data set consists of almost three years’ worth of data, collected every hour from 9 measuring stations in Czechia and Slovakia. Each sample in the data set represents a single signal gathered for 20 ms. The contactless method is deployed on the same stations as the galvanic contact method, which is used by power distributors and can provide ground truth. Also manually curated data by human expert are present. Successful detection of partial discharges can prevent electricity shutdowns and forest fires resulting from insulation failure due to vegetation contact. The data set is particularly relevant for covered conductors used in mountainous regions where establishing a safe zone is challenging. The contactless method offers advantages such as cheaper and easier installation. The data set has the potential to develop machine learning models to detect partial discharges and facilitate safer and cheaper use of covered conductors.

## Background & Summary

The problem with using covered conductors with XLPE material is high impedance faults, which are hard to detect (e.g., fallen tree). However, they can be detected by the presence of partial discharges (PD)^1^, which are both a signal and a harmful event. PD is a phenomenon that occurs in insulation materials used in power transmission systems, which can lead to insulation failure, power outages, and even fires^2^. The advantage of using CC in overhead power lines is its ability to withstand short contact with vegetation, making it useful for places where only a small safe zone can be maintained (e.g., national parks, hard-to-access terrains, etc.). This is useful to ensure the uninterrupted and reliable delivery of electrical power. When prolonged contact with vegetation happens, PDs (mainly surface PDs) start to appear and slowly degrade the insulation. Within a matter of hours or days (depending on the moisture of the vegetation and the environment), insulation failure occurs (as seen in Figs. 7, 8). For example, a very dry branch will not cause any PD and will pose no problem for CC. Insulation failure can cause accidents like a forest fire (Fig. 11) and others.

The main interest for power distribution companies is to detect if there is any contact with vegetation in order to prevent insulation failure. In the past, partial discharge detection for medium power voltage (22 kV) was performed mainly using galvanic contact method, which required physical access to the conductors and interruption of the power supply^3^. This method is used in production settings, for example in the Czech Republic and Slovakia, on over 22 sections of overhead power lines with CC. However, these method is rather expensive and time-consuming, and it require power outages for installation. Despite these drawbacks, it is used because it saves money and improves reliability. Therefore, alternative methods, such as the contactless antenna method, have been proposed to overcome these limitations^4^. This method is cheaper and does not require a shutdown of power during the installation of the detection device. On the other hand effective range of contactless method is smaller than contact method (1 km vs 4 km - depend on background noise in the measuring place) and acquired data is much noisier. Also as this method is cheaper (approx 10 times cheaper) more measuring stations can be deployed.

This study introduces a novel data set^5^ obtained using a contactless method for detecting partial discharges in XLPE-covered conductors used in medium voltage overhead power transmission lines^4^. The data set was collected every hour from nine measuring stations in the Czech and Slovak Republics, covering half a year of raw data. Each sample in the data set represents a single signal gathered for 20 ms. 20 ms are enough to capture single period as utility frequency is 50 Hz. The PD has a very distinct pattern in captured signal, which is used for distinguishing if PD are present or another type of like corona discharge is present. This data is from a real production environment on real power transmission lines with CC.

The primary goal of this data set^5^ is to facilitate the development of machine learning models that can detect partial discharges in covered conductors using the contactless antenna method. Successful detection of partial discharges can prevent electricity shutdowns and forest fires resulting from insulation failure due to vegetation contact^6^. The data set is particularly relevant for covered conductors used in mountainous regions where establishing a safe zone is challenging or where smaller safe zones are required.

The contactless method is deployed on the same stations as the galvanic contact method, which is used by power distributors and provides a ground truth. This allows for a comparison of the performance of the contactless method with the galvanic method. One of the inspirations for publishing this data set is a Kaggle competition^7^, which was held for the galvanic contact method and enabled the development of modern and very precise methods for the detection of PDs in CC. It is also one of the largest data sets available today to detect PD in CC^8–12^. Measuring stations are installed on poles with overhead power transmission lines for CC.

Several recent studies have used parts of this data set. Fulnecek *et al*.^4^ proposed, developed and tested a contactless fault detection system based on partial discharge activity detection for tree fall detection on MV overhead lines with covered conductors. Martinovic *et al*.^13^ proposed a fast algorithm for contactless partial discharge detection on a remote gateway device, which consists of outlier detection, clustering, feature extraction, and classification. Klein *et al*.^14^ introduced a machine learning algorithm based on a heterogeneous stacking ensemble neural network to classify partial discharges obtained by a contactless detection method using an antenna. They also explored the ability of edge computing devices, such as the Jetson platform, Neural stick, and Edge TPU, to solve the real-world problem of detecting partial discharges from covered conductors on high-voltage power lines located in remote and heavily forested areas^15^. None of these studies had published a data set.

These recent studies show promising results and highlight the potential of this data set to develop machine learning models to detect partial discharges. This, in turn, can facilitate safer and cheaper power transmission using covered conductors.

The data set has the potential to improve the accuracy and efficiency of detecting partial discharges in covered conductors and facilitate safer and cheaper power transmission. It has the potential for reuse in various applications, including fault diagnosis, power system protection, and predictive maintenance. In addition, the data set can be used to evaluate the performance of different machine learning models and compare them with traditional galvanic contact methods.

## Methods

To obtain the data set, data acquisition devices were installed alongside an existing galvanic contact method. The galvanic contact method provides ground truth for detection and reliable data to compare, while contactless partial discharge detection involves the use of an antenna sensor. The antenna sensor can be easily installed on existing medium voltage (MV) overhead lines without modifications to the covered conductor (CC) and without the need to disconnect the line, making it a convenient and cost-effective option as it it much cheaper then using contact galvanic method. Moreover, the antenna sensor only requires a single channel DAQ, which reduces the requirements for wireless data transmission. Unlike the galvanic contact sensor, the antenna sensor does not require an MV capacitive divider for its operation, which is expensive. Most common XLPE CC cables used in transmission lines, where measuring station are installed are 1AS or CCX WK 20Kv. The description of used cables can be found for example on website of manufacturer PRAKAB PRAŽSKÁ KABELOVNA s.r.o. (https://www.prakab.cz/en/products/insulated-overhead-lines/).

### Measuring platform

As the hardware was reused, the Remote Terminal Unit (RTU) used for the galvanic contact method was used as there was enough free computation power and a last free data acquisition channel with a 8 bit ADC that has a very high sample rate and variable range up to 1 Vpp (Voltage peak to peak). The hardware of the detection device is a proprietary solution manufactured by the ELVAC company. The detection devices contain ARM CPUs and run the Linux operating system.

The measuring platform for PD detection was mounted on the single pole of an overhead line, consisting of a box containing a power source with a backup battery, a DAQ with a sample rate of 40 MS/s (Mega sample per second), and a control unit. The antenna and contact method can be seen in Fig. 10. The signals were then examined in a frequency spectrum of up to 20 MHz, as this was the maximum for the DAQ sample rate (according to the Nyquist-Shannon sampling theorem). This means that the range of frequencies acquired was from 20 kHz to 20 MHz. As a common type of wideband whip antenna with compact dimensions and low price is used, the Boni-Whip antenna. The antenna is mounted on the pole, parallel to the CC.

### Antenna sensor

The Boni-Whip antenna is a type of active wideband whip antenna with a frequency range of 20 kHz - 300 MHz We only use frequencies up to 20 MHz that is commonly utilized as an antenna sensor. It is a compact, cost-effective, and highly portable device that can be easily installed on overhead lines. This antenna is an improvement on the successful Mini-Whip antenna and is developed and manufactured by Bonito, a German-based company.

The Boni-Whip antenna provides excellent reception results on long-wave, medium-wave, short-wave, and VHF, making it ideal for a wide range of applications. Its gain is 3 dB, and its upper frequency limit is (−1 dB): 300 MHz, with IP3 of +32.5 dBm and IP2 of +55 dBm. The voltage supply for the antenna is delivered by the antenna cable and the included power supply module (bias tee).

The antenna sensor requires a nominal power supply voltage of 12 V with a maximum current draw of 150 mA. To power the entire acquisition platform, an instrumental transformer (22/0.1 kV) is utilized, which is directly connected to the power line under examination. However, the amplifiers within the DAQ platform introduce a DC offset in the acquired signals. Fortunately, the amplitude of this offset is typically very small, and thus it rarely results in any substantial issues.

It should be noted that the sensitivity of the BONI whip antenna sensor depends on various factors, such as the length, height above ground, type of conductor, and actual grid configuration of the power line. Under laboratory conditions with an overhead power line model connected to the PD calibrator, the sensor sensitivity was found to be 1 nC/10 mV. However, in real-world environments, this value is subject to variability. On-site calibration is the only reliable method for determining the sensitivity of the sensor in such conditions. Unfortunately, conducting on-site calibration would require the entire distribution power grid to be shut down, which is not feasible for distribution grid operators. As a result, no on-site calibration has ever been performed.

### Sample acquisition

**Fig. 1:**
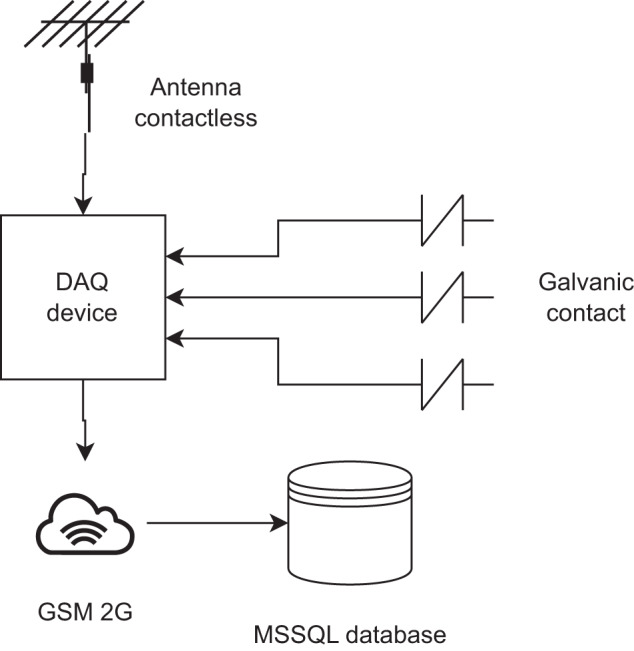
Data collection process.

The acquired signal comprises 800,000 samples, representing a single period of the frequency of the power grid. The sampling is performed every hour and the data is transmitted remotely using 2 G GSM technology. Remote stations are often located in remote or inaccessible areas, where the 2 G signal is unreliable. Thus, transmitting additional samples is not feasible. Furthermore, in the event of a tree fall, PD activity remains stable and continuous, resulting from the tree’s direct contact with the covered conductors, posing a severe threat to the conductor insulation. Although unstable PD activity is common due to random contacts with surrounding vegetation, such events have little impact on insulation damage. On the basis of our experience, hourly data acquisition is sufficient for detecting insulation damage. The general information on data acquisition can be seen in Fig. 1.

### Data retention

The data retention process involved storing the collected data in a Microsoft SQL Server database. The data were saved in the image data type that contained the measured signal information. Additionally, metadata such as station, phase, date and time, and operator details were stored in accompanying tables or columns.

To optimize the data storage process, the data was imported as batches as soon as it was gathered by the distribution operators. However, the frequency of data transfer varied among different operators, with some sending data consistently, while others did so infrequently.

The stored data was regularly classified and verified to ensure its quality and relevance to the research objectives. This process helped filter out any irrelevant or corrupted data, which could have negatively impacted the accuracy of the results. In general, the data retention process was critical in providing a reliable and comprehensive data set.

As the provided dataset is related to work on national infrastructure, critical data was anonymized to ensure the safety of infrastructure (location, etc.). Also, the primary purpose of gathering this kind of data is to ensure more reliable and safer use of XLPE-covered conductors.

### Signal description

The signal measured by the wireless antenna contains a large amount of noise artifacts, mostly generated by the AM radio and the resistance in the circuit itself. The device always measures the highest received frequency, and noise usually hides a large part of the original signal, creating an almost compact body when visualized. This compact body changes its magnitude over time, making it impossible to define a single value that would discern outliers and noise.

The signal is processed by an 8-bit analog-to-digital converter (ADC), which returns values in the range from −128 to 127. A signal plotted as amplitude over time usually contains two larger clusters of higher electromagnetic activity, corresponding to the three phases of an alternating current in overhead power lines. Visible peaks in the signal may indicate the presence of partial discharges or other noise.

The signals acquired in a real environment contain high levels of noise, unlike in laboratory conditions. There are various sources of background noise, including radio broadcasts, atmospheric disturbances, switching power supplies, and other types of discharges. Based on the influence of background noise on the frequency spectrum and time domain of acquired signals, most noise signals in observed data can be separated into two groups: discrete spectral interference (DSI) and random pulse interference (RPI)^4^.

Additionally, the data contains a large amount of noise, as well as time series associated with other types of discharge, such as corona discharge (corona discharge has different pattern^6^) or rime on covered conductors (still unsolved problem as rime has very similar characteristics to PD. These discharges have different frequency characteristics, which make them distinguishable from partial discharges.

In conclusion, the acquired signal from the wireless antenna contains a complex mix of partial discharges and background noise, which requires careful processing and analysis to detect and distinguish between them accurately.

### Fault annotation using galvanic contact method

In addition to measuring signals to detect partial discharge (PD) using the antenna, a galvanic contact method is installed alongside for the annotation of faults. This method is used for supervised training to detect faults using the detection of PD. The annotation is done by an unpublished but production-used algorithm, which is utilized by Czech energy distributors.

The galvanic contact method has been shown to have high accuracy in fault detection with a precision of 70%, sensitivity of 98.4%, specificity of 99.8%, and an overall accuracy of 96.7%^13^. However, it should be noted that the effective range of contactless detection is shorter than that of the galvanic contact method, with the latter having a range of 1 km compared to 4 km for the former. The effective range is also dependent on the environment and background noise^4^.

### Manually annotated subset

A manually annotated subset was created as an alternative to the use of the galvanic contact method as labels. This subset was created through a meticulous data selection and validation process by an expert on partial discharges (PDs). The expert reviewed the data and confirmed the visible appearance of the PDs or the lack thereof (See Figs. 5, 6 which do not have any PD presence). As the PD pattern is very distinctive^4^. (See Figs. 5, 6 which contains PD pattern).

To ensure accurate labeling of the time series, a human operator checked all samples for the presence of partial discharges using the galvanic method of detection of PD. The selected samples were diverse in their characteristics, including samples without PD that contained other types of discharge, such as corona. This diversity of samples should help the model learn to distinguish between PD and other types of discharge.

Although this approach allows for a high level of precision in identifying PDs, the major drawback of this method is that the size of the annotated subset is limited. However, the advantage of this method is that it provides a high level of accuracy in identifying PDs without the need for additional hardware or equipment, making it a more cost-effective solution.

It should be noted that the annotated subset provides a reliable ground truth for supervised learning, allowing the training of machine learning models for the detection of PD. Additionally, this subset can be used to evaluate the performance of PD detection algorithms and compare their accuracy with the results obtained from the galvanic contact method.

## Data Records

**Fig. 2:**
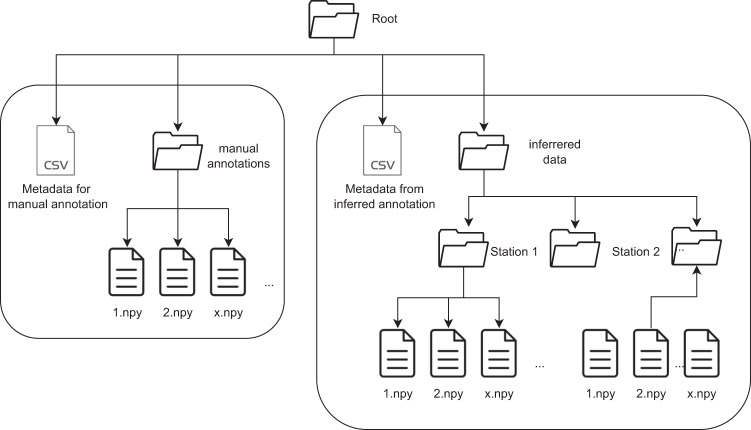
Overview of folder hierarchy.

We have uploaded our dataset to fiqshare system^5^. A hierarchical folder structure is used to ensure efficient and organized data management. At the root level, two CSV files store metadata and annotations related to measurements. One is for manually validated data (manual_annotation.csv) and second one is for data with inferred annotation from the galvanic contact method (inferred_annotation.csv). An overview of the hierarchy folder can be seen in Fig. 2.

There are directories for each station, where the acquired signals are stored. Samples for each station are saved as numpy arrays in .npy format. Each sample is identified by a measurement ID, and the path to each sample is the station ID followed by the measurement ID, which is also the filename of the sample.

A single folder (manual_annotation) is used to store the manually validated data set, which includes signals selected by experts based on their experience and knowledge of PD behavior. The selected signals are carefully examined and annotated, and only those meeting high-quality criteria are included in the data set. There is a separate metadata file for this data (manual_annotation.csv).

The data are stored in a compressed format to optimize storage space, and the compression rate is very high due to the large size of the data files. The data for each station and the whole data set^5^ folder was compressed using the tar -cJf command, which creates a compressed tarball archive file in the .tar.xz format. This format is known for its high compression ratio and is commonly used to compress large files or directories.

This hierarchical folder structure ensures that the data is well-organized and easy to navigate, facilitating data analysis and further research. High compression rates are used for efficient storage of the large amount of data acquired, while the careful selection and annotation of the manually validated data set^5^ ensures its high quality and reliability.

### Metadata description

There are two metadata files for the samples, one for manually annotated data (manual_annotation.csv) and another for data with annotations from the galvanic contact method (inferred_annotation.csv). Both metadata files are in CSV format with a comma delimiter, and the first row is the header.

The metadata file for annotations from the galvanic contact method contains a unique measurement ID used for internal identification, the station ID where the measurement was performed, the index in the array saved in the file for the given station, a timestamp in ISO 8601 format indicating when the measurement was taken, and fault annotation from the galvanic contact method.

The metadata file for manually annotated data follows a structure similar to the galvanic contact method, but does not include a timestamp. Expert annotations on partial discharges were used instead.

### Description of samples

**Fig. 3:**
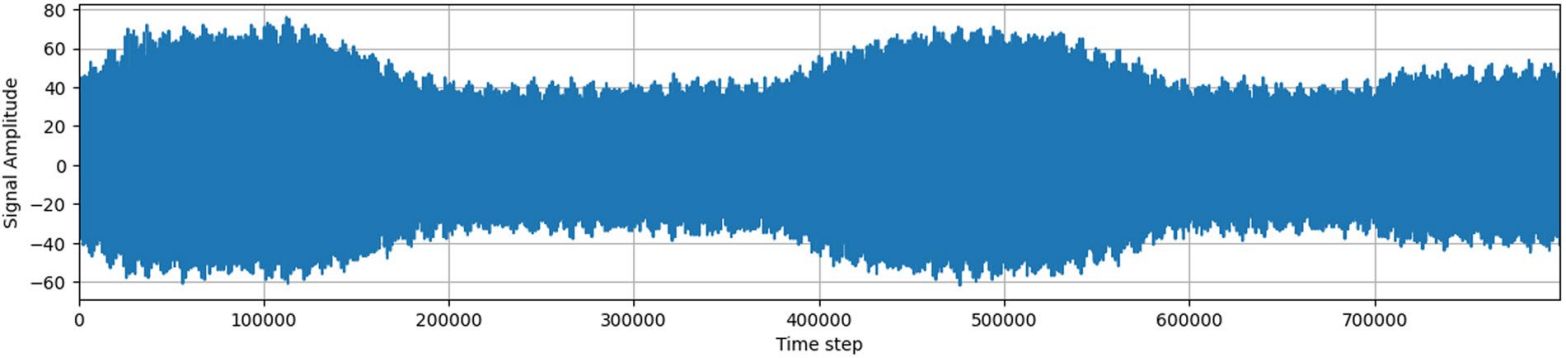
Example of an acquired signals without PD pattern.

**Fig. 4:**
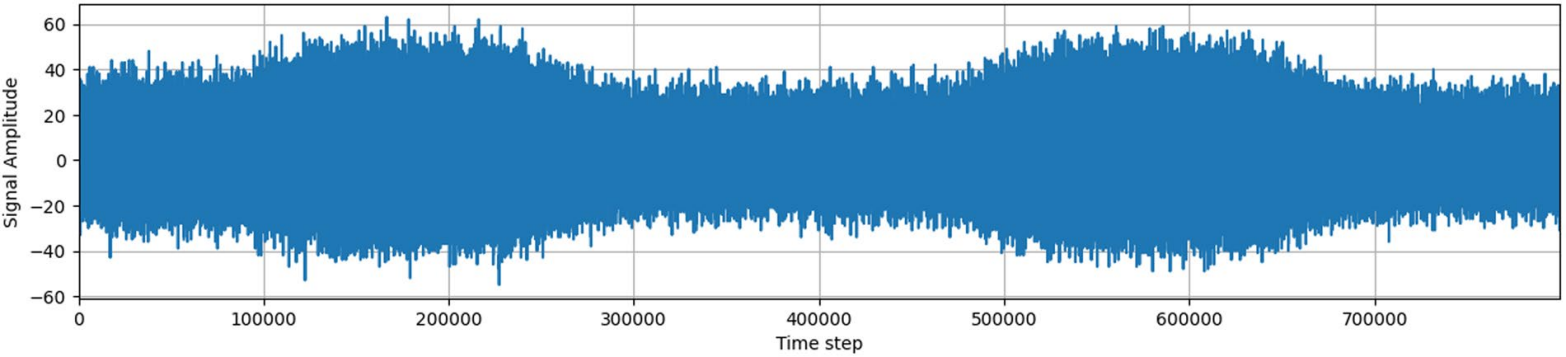
Second example of an acquired signals without PD pattern.

**Fig. 5:**
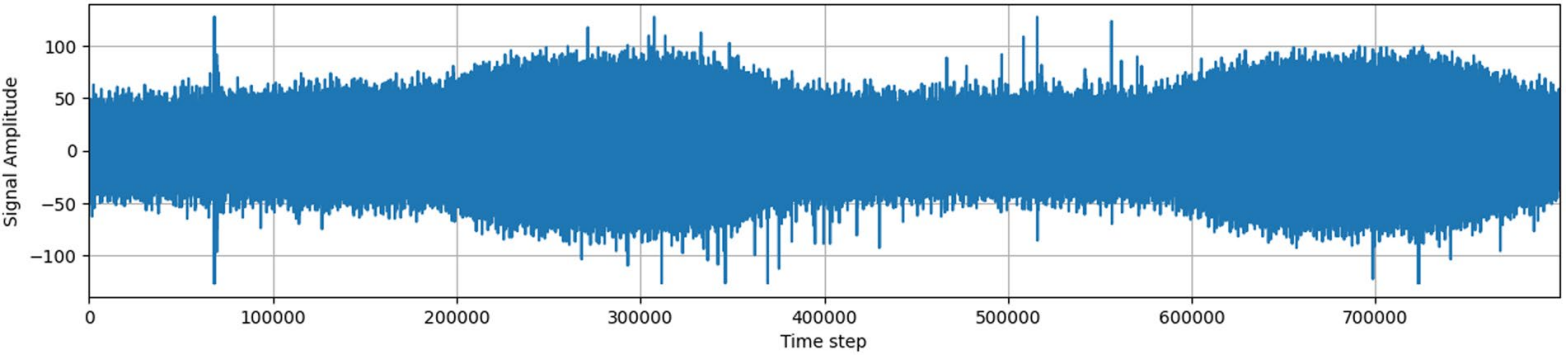
Example of an acquired signals with PD pattern.

**Fig. 6:**
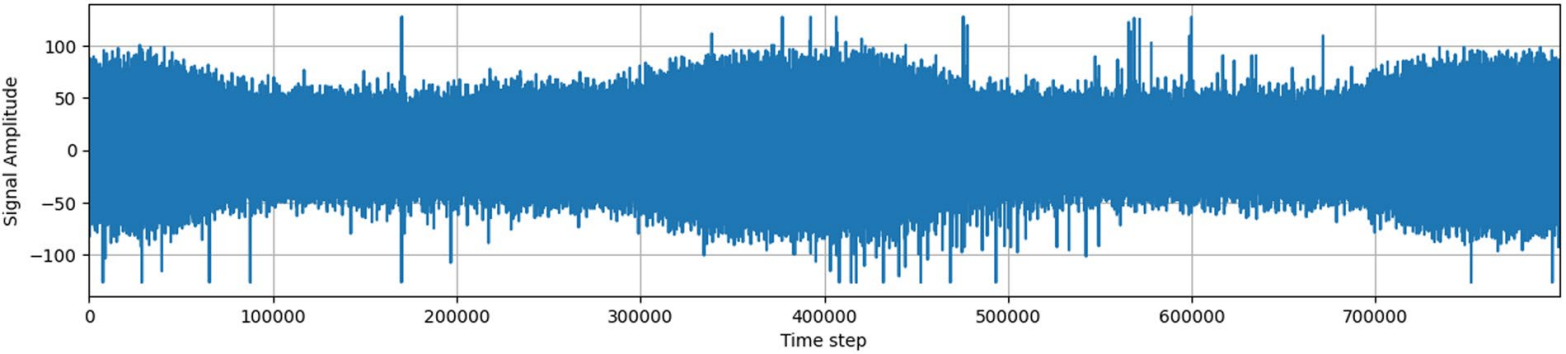
Second example of an acquired signals with PD pattern.

Samples for each station are saved as numpy arrays in a .npy format. Each sample has a length of 800,000 and a signed 8-bit data type (numpy dtype int8). Figs. 3–6 show a sample of the signal. The filename of the sample is a measurement ID and each sample is in directory with a name of station or for manually annotated data in directory manual_annotation.

**Fig. 7:**
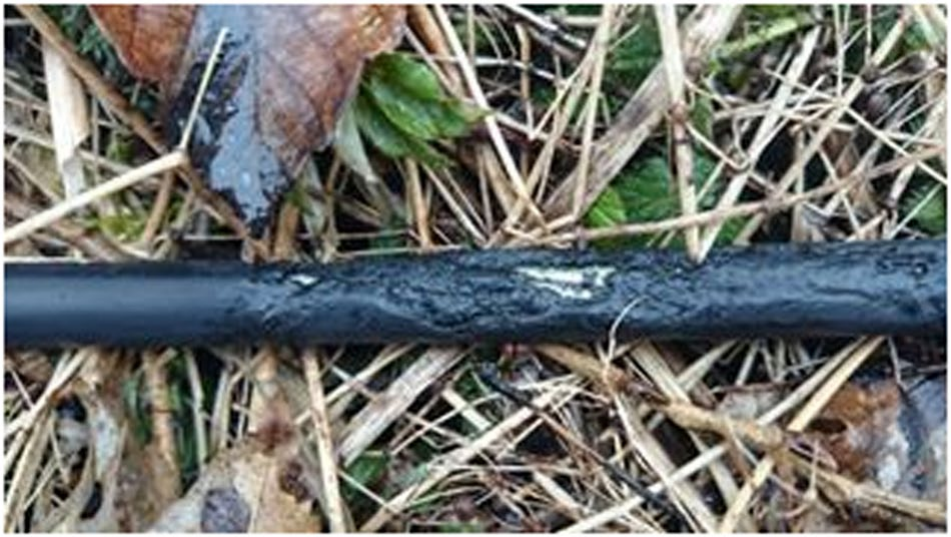
Example of damaged insulation after prolonged contact with vegetation (tree).

**Fig. 8:**
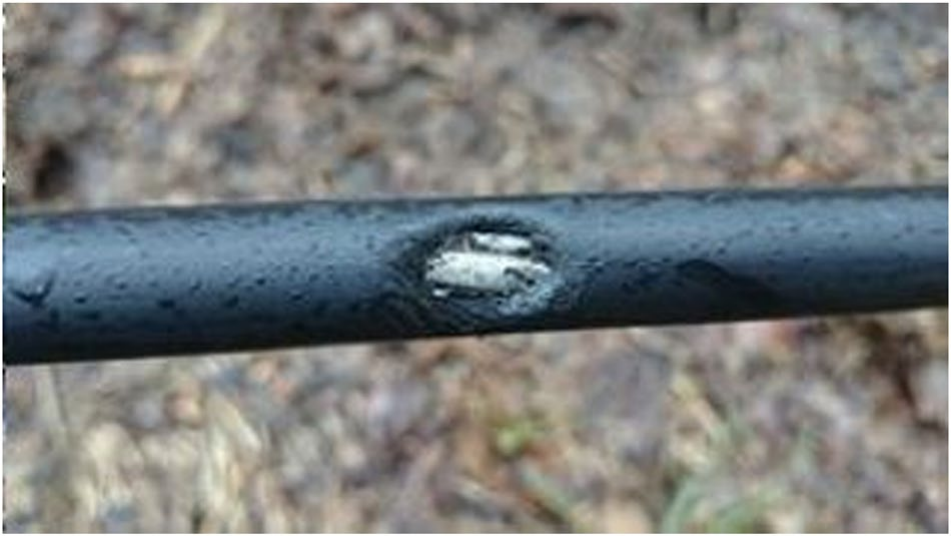
Second example of damaged insulation after prolonged contact with vegetation (tree).

The ID used for internal identification is consistent and can be used to match measurements from previously published galvanic contact method data sets^7^. A new data set^5^ containing a large amount of data for galvanic contact measurements will also be published, along with a new classification method for the contact galvanic method, by another team. We have no influence on when it will be available or published.

Table 1 shows the sample count in the data set^5^ for cases with and without PDs, as well as the total count, for both manual and inferred annotations.

**Table 1:** The data set sample count.

	Without PDs	With PDs	Total
Manual annotation	1520	180 (10%)	1700
Inferred annotation	149493	1941 (1.2%)	151434

## Technical Validation

Besides continuous validation and use of data for the research team and the development of new machine learning algorithms, we had performed a technical validation of the provided data set.

To ensure the quality and accuracy of the data^5^, a thorough technical validation was performed. This validation involved a combination of visual checks, statistical analysis, and spot testing of random samples.

First, the data were visually checked by station and ordered by time. For each station, every 1000th sample was examined to ensure that the data were consistent and free from errors or anomalies. Additionally, a random 1% of the data was also checked to ensure that the validation process was thorough and comprehensive. We provide script to validate data by reader - test_station.py.

Furthermore, to ensure the accuracy of the data^5^, 100 samples with PDs were selected and checked. Additionally, ten random samples per station were also examined to ensure that the data were consistent across all stations - test_fault.py and test_station.py.

To further validate the data^5^, statistical analysis was performed on the data for each month. This involved calculating various statistical measures, such as means, medians, and standard deviations, for each station and comparing the results to expected values. Any discrepancies or anomalies were investigated and resolved to ensure the accuracy and integrity of the data. This is in the provided script - test_monthly.py.

Data acquisition is essential for capturing signals for further analysis. However, due to a variety of factors such as problems with the data acquisition device (DAQ) or connectivity issues, data acquisition is not always continuous. Therefore, the intervals between the data points are checked for consistency and absence of errors or anomalies to ensure data accuracy. This is crucial as missing values or gaps can potentially impact the integrity of the data.

Although missing values or gaps in the data may exist, they do not necessarily invalidate the data. Each sample is independent and sufficient for classifying the presence of partial discharge (PD) faults. However, if the samples are to be used as time series, missing data should be taken into account.

In our analysis, we provide a script (intervals.py) to retrieve continuous samples and visualize gaps in data acquisition. Our results show (in Table 2) that all stations experienced gaps in data acquisition, with the majority being single missing samples. Interrupts greater than 24 hours were rare, with station 52010 having the most significant gaps in data acquisition. This station also had a limited amount of data, contributing to the gaps in data acquisition.

**Table 2:** Table of number of interrupted intervals in data by time period and station ID.

Station ID	Interrupted Intervals				
	1 Hour	8 Hours	24 Hours	72 Hours	One Week
52007	436	31	17	5	2
52008	196	15	11	6	4
52009	473	30	22	9	6
52010	542	119	72	38	22
52011	191	21	17	10	8
52012	304	18	15	6	4
52013	571	59	12	7	5
52014	1090	66	18	8	4

**Fig. 9:**
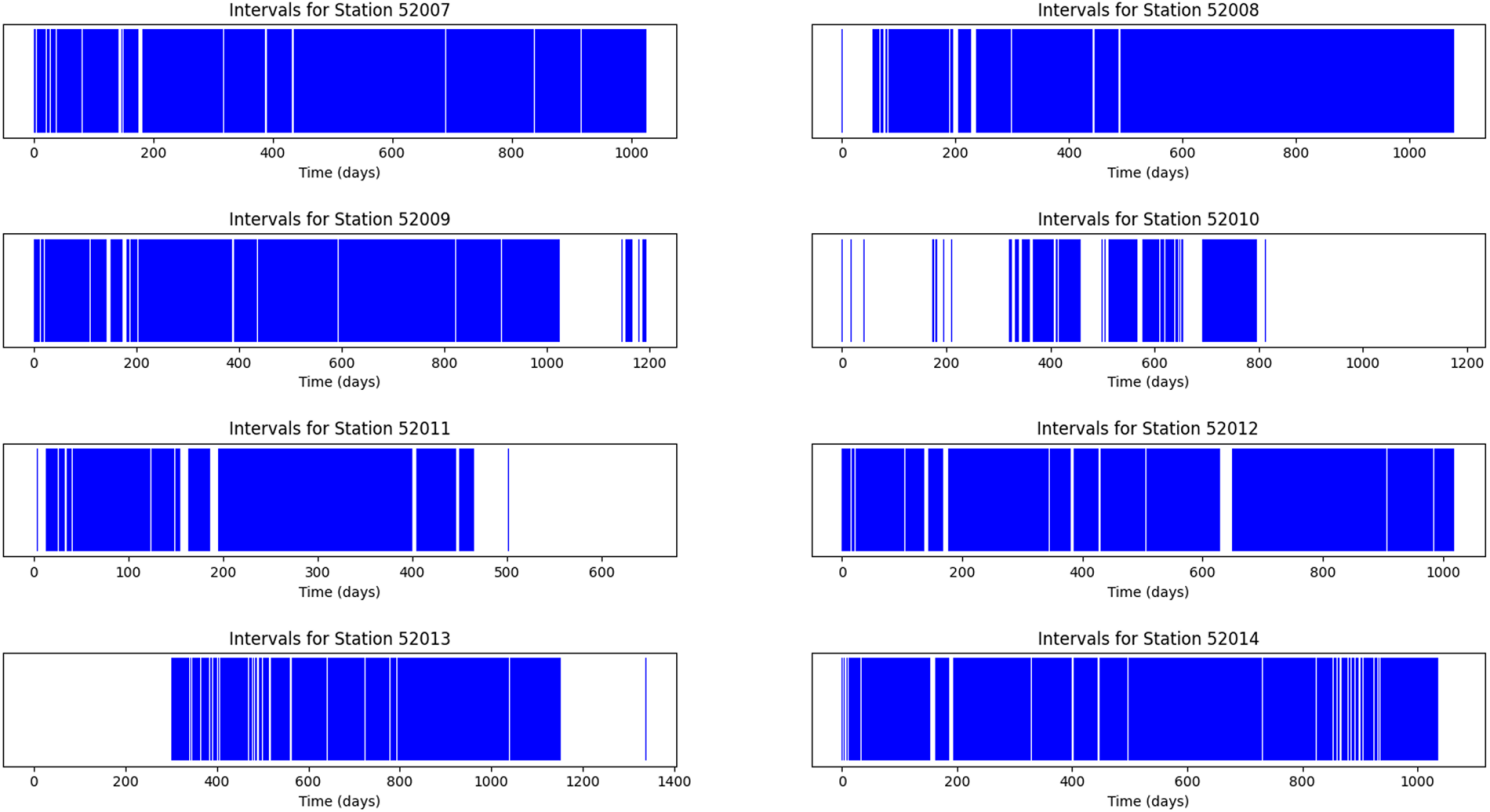
Temporal distribution of data samples.

Figure 9 provides an overview of the missing data intervals for each station. Station 52007 and 52008 had the most complete data acquisition, while other stations had gaps in data acquisition. It is worth noting that maintaining stable connectivity and functionality is not always possible in the monitored environment and locations.

**Fig. 10:**
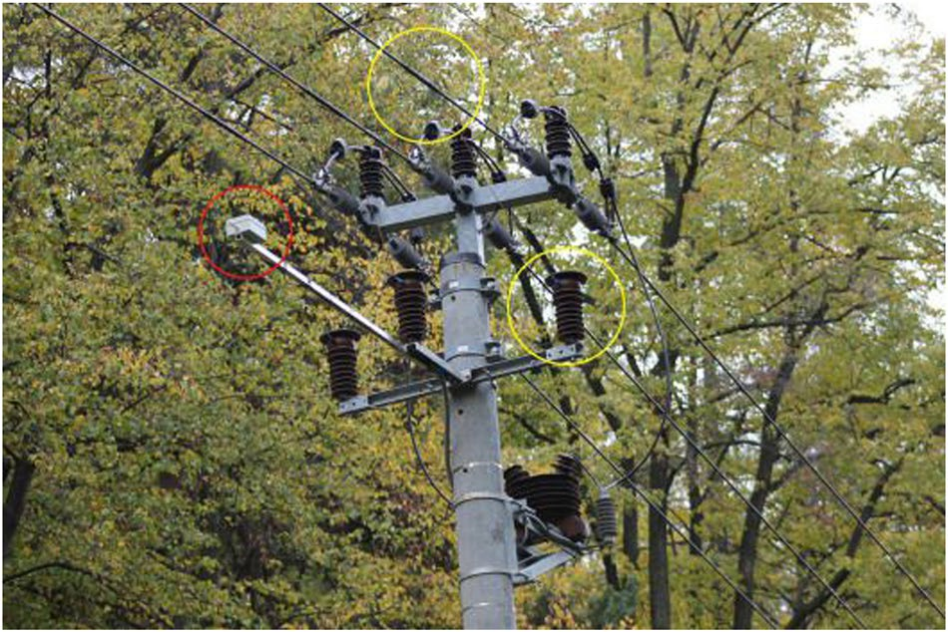
Single pole with installed a galvanic contact method (yellow circles) and antenna based method (red circle).

**Fig. 11:**
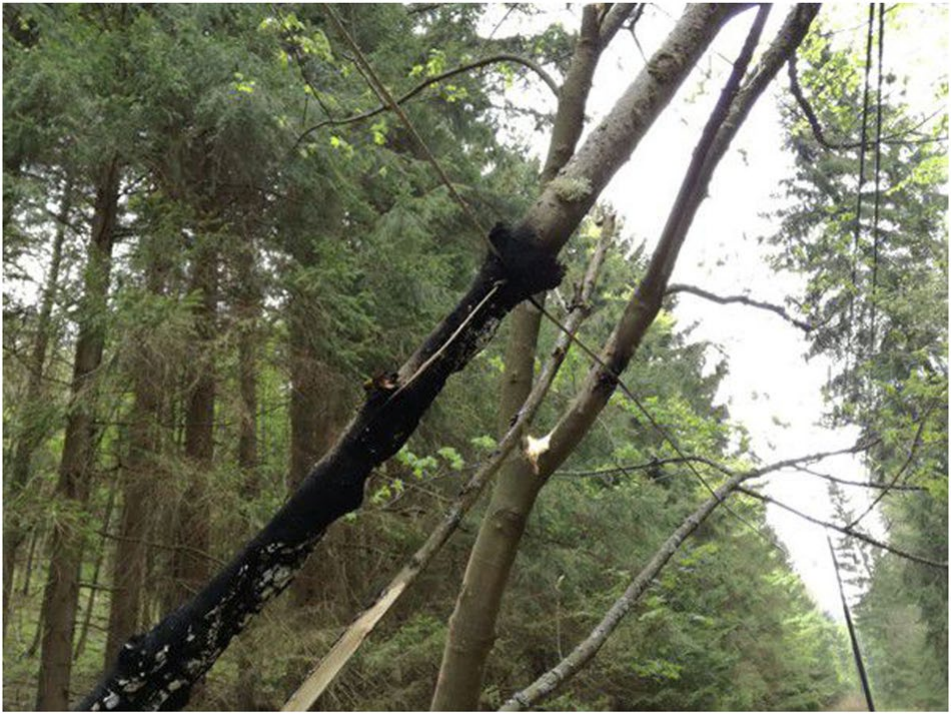
Example of a burned tree after insulation failure.

In general, the technical validation process confirmed the quality of the data^5^, and we are confident that it can be used with confidence by researchers and data scientists alike. The data set^5^ has the potential to be reused in various applications, including fault diagnosis, power system protection, and predictive maintenance. Furthermore, the data set^5^ can be used to evaluate the performance of different machine learning models and compare them with traditional galvanic contact methods. In Table 3 can be seen a distribution of samples between stations and number of samples with or without PDs.

**Table 3:** Samples per station for inferred data.

Station ID	Without PDs	With PDs	Total
52007	22700	269	22969
52008	22628	998	23626
52009	23477	164	23641
52010	8161	63	8224
52011	10007	9	10016
52012	22757	5	22762
52013	18235	353	18588
52014	21528	80	21608

## Usage Notes

The data provided is in the npy format, which is the standard binary file format used in NumPy to persist a single arbitrary NumPy array on disk. This format stores all the necessary shape and dtype information to correctly reconstruct the array, even on a different machine with a different architecture.

In Github repository there is also a script to extract all data from compressed archives.

To work with these data, it is recommended to use the NumPy library, which provides efficient and flexible data structures for numerical computation. In addition, since the data files may be large, it is recommended to use tools such as Dask or memmap to handle these files.

Dask is a library for parallel computing in Python that allows for out-of-core computation on large data sets. It can be used to handle data that is too large to fit into memory by partitioning the data into smaller chunks and processing them in parallel.

Memmap is a feature in NumPy that allows for memory-mapped files, enabling efficient access of large arrays stored on disk. By using memory-mapped files, only the portions of the array that are accessed are loaded into memory, which can greatly reduce memory usage.

Metadata for the data is provided in csv files, which can be easily read and manipulated using tools such as pandas or NumPy.

## Data Availability

The code for loading the data set^5^, testing, and utility functions is available in a Github repository, which can be found at the following https://github.com/Lukykl1/dataset_pd_vsb. The repository includes code for loading the data in npy format and the metadata in csv format, as well as utility functions for processing and analyzing the data. The code is documented and includes comments to explain each step of the process. To run the Python scripts, you will need to have the necessary libraries installed, including NumPy, pandas, Matplotlib, and any other required dependencies. You can install these libraries using a package manager such as pip or conda. We hope that this code will be useful to researchers and data scientists who are working with these data and looking for an efficient and flexible way to load and process them.
